# Optical coherence tomography classifications of diabetic macular edema and response to aflibercept: One-year follow-up outcomes in a Chinese population

**DOI:** 10.1097/MD.0000000000032815

**Published:** 2023-01-27

**Authors:** Zheyao Gu, Ting Xi, Chunyuan Zhang, Guang Yang

**Affiliations:** a Department of Ophthalmology, The Affiliated Suzhou Hospital of Nanjing Medical University, Suzhou Municipal Hospital, Suzhou, China.

**Keywords:** anti-vascular endothelial growth factor (VEGF) treatment, best corrected visual acuity (BCVA), central macular thickness (CMT), diabetic macular edema (DME), optical coherence tomography (OCT)

## Abstract

To evaluate the effect of intravitreal aflibercept on different classifications of diabetic macular edema (DME) by spectral-domain optical coherence tomography. This hospital-based retrospective study included 95 consecutive patients (130 eyes) diagnosed with DME. Three groups were defined: diffuse retinal thickening (DRT), cystoid macular edema and serous retinal detachment. All eyes received intravitreal aflibercept (0.05 mL/2 mg) 5 times monthly. Best corrected visual acuity (BCVA) in (logarithm of the minimum angle of resolution) units and central macular thickness (CMT) on optical coherence tomography were recorded at months 1, 2, 3, 4, 6, and 12 after the injections. There was no significant baseline difference in BCVA (*P* = .273) or CMT (*P* = .115) among the 3 groups. Over 12 months, the BCVA of the DRT group significantly improved from baseline (*P* = .013). The BCVA of the cystoid macular edema (*P* = .062) and serous retinal detachment groups (*P* = .073) improved slightly from baseline. The DRT group had the greatest BCVA improvement (*P* = .021). Over 12 months, the CMTs of all 3 groups significantly decreased from baseline (*P* = .016, *P* = .025, *P* = .031). The CMT decreased more in the DRT group than in the other 2 groups (*P* = .009). The CMT changes were most evident in the DRT group (*P* = .022). Binary logistic regression analysis showed that DME type, disorganization of the retinal inner layers, ellipsoid zone disruption and external limiting membrane disruption independently predicted the effect of aflibercept treatment in DME patients (*P* = .006, *P* = .001, *P* = .004, *P* = .001). Aflibercept therapy improved anatomical structure and visual acuity in every type of DME; DRT responded best in terms of both BCVA and CMT. Furthermore, DME, disorganization of the retinal inner layers, external limiting membrane disruption and ellipsoid zone disruption independently predicted the effect of aflibercept treatment in DME patients.

## 1. Introduction

Diabetic macular edema (DME) is one of the common complications of diabetes mellitus and a major cause of blindness in diabetic patients.^[1]^ According to the International Diabetes Federation, the overall number of diabetic patients globally reached 425 million in 2017 and will reach 629 million by 2045, with the prevalence of DME in adults being approximately 7.6%.^[2]^

The formation of DME is mostly related to increased retinal capillary permeability, breakdown of the blood–retinal barrier, and local retinal thickening and edema.^[3]^ At present, the treatment of DME includes laser therapy, intravitreal corticosteroids, intraviral anti-vascular endothelial growth factor (VEGF), and surgical treatments.^[4]^ Among them, intravitreal injections of anti-VEGF drugs have been the main treatment for DME. It has been confirmed that anti-VEGF drugs can specifically inhibit the expression of VEGF in local tissues and effectively reduce DME by inhibiting the generation and progression of neovascularization and improving the permeability of retinal and choroidal microvessels.^[5]^ Currently, there are many kinds of anti-VEGF drugs used worldwide as first-line therapy for the treatment of DME, such as bevacizumab, ranibizumab, and aflibercept.

Aflibercept (Eylea^@^, Regeneron Pharmaceuticals, NY) is a 115 kDa recombinant fusion protein consisting of the VEGF receptor with 2 protein segments (VEGFR-1 and VEGFR-2) and the fragment crystallizable of human immunoglobulin G1.^[6]^ It has a strong affinity for VEGF-A, VEGF-B, and placenta growth factor. Therefore, it can inhibit not only the pathological effects of VEGF in DR but also placenta growth factor.^[7]^ The VIVID, VISTA, and VIVID-EAST studies all confirmed that intravitreal aflibercept can significantly improve the visual acuity of DME patients, especially for people with low vision.^[8]^

Intravitreal anti-VEGF drugs have been found to be effective in the treatment of DME. However, it has been suggested that DME diagnosis and management remain lacking in many patients with diabetes, particularly in China. Therefore, the diagnosis and staging of patients with DME are particularly important. Spectral-domain optical coherence tomography (OCT) is a standard noninvasive test used to diagnose and evaluate DME. According to the different OCT characteristics, DME is mainly classified into diffuse retinal thickening (DRT), cystoid macular edema (CME), and serous retinal detachment (SRD).^[9]^ It has been identified that the efficacy and prognosis of anti-VEGF drugs vary among different types of DME patients.^[10]^ At present, most contemporary research studies have focused on anti-VEGF drugs such as bevacizumab and ranibizumab. However, there are few studies on the effectiveness of aflibercept in treating different types of DME.

In this retrospective study, we analyzed the effect of aflibercept on the treatment of DME patients with different OCT types, aiming to better assess the treatment effect of different types of DME and provide a reference for the clinical treatment of DME patients.

## 2. Methods

### 2.1. Ethics statements

The study adhered to the principles of the Declaration of Helsinki and was approved and agreed upon by the ethics committee of Suzhou Municipal Hospital. (Project No. KL901177). All consecutive patients were informed of the risks associated with surgery and signed the surgical consent form before undergoing the procedure.

### 2.2. Study design and patient selection

A hospital-based retrospective study was included at an ophthalmology department (Suzhou Municipal Hospital, Suzhou, China) from September 2020 to December 2021. Patients in this study were given aflibercept injections at 1-month intravitreal injection 5 times, with a 12-month follow-up. The analysis comprised a total of 130 eyes that matched the eligibility criteria.

#### 2.2.1. Inclusion and exclusion criteria.

The following were inclusion criteria: Over 18-year-old individuals with type 2 diabetes mellitus diagnosed by the endocrinology department; meeting EURETINA diagnostic criteria for DME; patients with central macular thickness (CMT) > 300 μm^[11]^ by spectral-domain-OCT used for the commercially available Cirrus HD-OCT Model 5000 (Carl Zeiss Meditec, Dublin, Ireland) (the mean CMT was automatically generated and measured in the central 1 mm; according to fundus fluorescein angiography grading, all eyes with DME were in the nonproliferative diabetic retinopathy stage); and willing to receive anti-VEGF treatment and able to receive regular follow-up.

The following were the exclusion criteria: Refractive interstitial clouding affecting fundus observation; combined retinal pathology such as vitreous hemosiderosis and retinal fibroplasias; other fundus disorders that can induce macular edema, such as age-related macular degeneration and retinal vein obstruction; and preoperative optic nerve and other retinal pathologies affecting visual function.

### 2.3. Evaluations, treatment, and data collection

At baseline, all consecutive patients underwent a comprehensive ocular examination, which included best corrected visual acuity (BCVA) testing, intraocular pressure (IOP) measurement, color fundus photography, dilated fundus examination with slit-lamp biomicroscopy, and fundus fluorescein angiography. BCVA was measured using an international standard visual acuity chart and converted to logarithm of the minimum angle of resolution (logMAR) visual acuity for data analysis. The clinical information of the consecutive patients, including age, sex, duration of diabetes, and glycated hemoglobin (HbA1c), was collected.

Changes in logMAR BCVA and CMT as measured by OCT were the major outcome measures. Follow-up examinations were performed every 1 to 2 months after 5 intravitreal injections, depending on the patient’s condition. BCVA evaluations, CMT measured by OCT and fundus exams were performed, and data gathered immediately following each injection, as well as 6 and 12 months afterwards, were evaluated. During the follow-up time, when visual acuity loss was >5 letters or CMT increased by >10%, additional injections were performed.

#### 2.3.1. OCT classification of DME.

**Figure 1.**
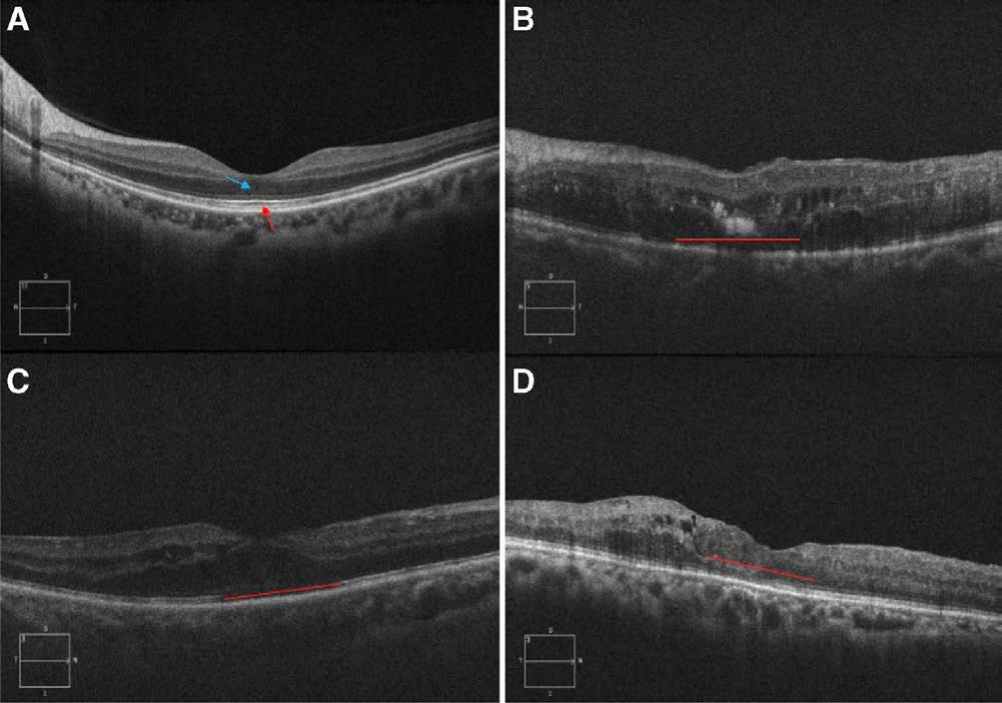
Representative SD-OCT images. (A) Horizontal OCT image of the retina of a normal eye showing the locations of the ELM and EZ. In the figure, the red arrow is the EZ, and the blue arrow is the ELM. (B) The horizontal length of ELM disruption in the 1-mm central area. (C) The horizontal length of EZ disruption in the 1-mm central area. (D) The horizontal length of DRIL in the 1-mm central area. DRIL = disorganization of the retinal inner layers, ELM = external limiting membrane, EZ = ellipsoid zone, SD-OCT = spectral-domain optical coherence tomography.

OCT scans were performed from dilated pupils. The central macular recess is the center of the scan, and the scan length is 6 mm, which can be adjusted according to the size of the lesion. The resolution was 5 μm, the scan depth was 4 mm, and the scan mode was a 512 × 128 horizontal linear scan performed by HD-OCT (Cirrus HD-OCT Model 5000; Carl Zeiss Mediatic Inc, Dublin, CA) to measure retinal thickness at the central fovea and identify DME based on the morphologic pattern. Disruption of the ellipsoid zone (EZ) and external limiting membrane (ELM) was assessed within 1 mm of the horizontal scan line of the central fovea. The presence of disorganization of the retinal inner layer (DRIL) was evaluated within the central 1 mm region using the OCT images (Fig. 1).

**Figure 2.**
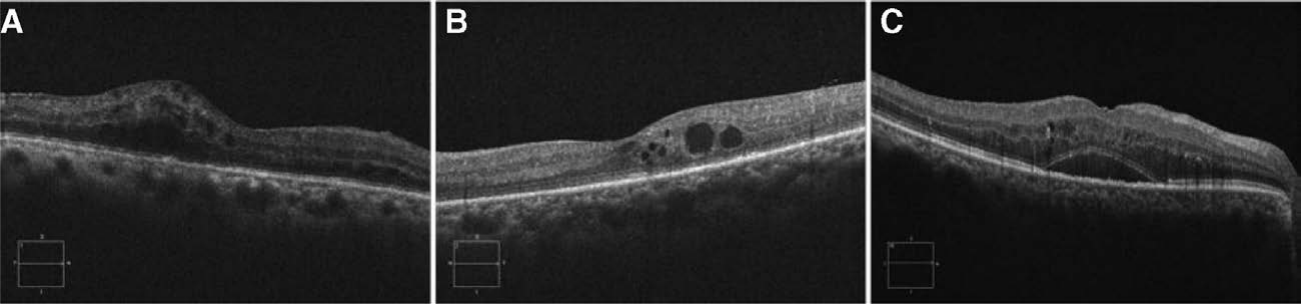
Different types of DME by OCT. (A) DRT type: sponge-like swelling of the retina. (B) CME type: several dark areas containing fluid cysts in the macular retina. (C) SRD type: a relatively transparent liquid dark area between the neuroepithelium and the retinal pigment epithelium. CME = cystoid macular edema, DME = diabetic macular edema, DRT = diffuse retinal thickening, OCT = optical coherence tomography, SRD = serous retinal detachment.

DME was classified into 3 groups. The DRT type was characterized by widespread retinal thickening with sponge-like hyporeflective edema of the macula. The CME type was characterized by the formation of macular edema followed by the appearance of regularly spaced hyperreflective stripes, leading to a focal mound-like area of hyporeflective edema in the macular area. The SRD type was characterized by an elevated neuroepithelium and a relatively transparent liquid dark area between the neuroepithelium and the retinal pigment epithelium (RPE) (Fig. 2). If both DRT and CME are present, the OCT image selects the most dominant mode. If neither of these patterns was predominant, the eye was not enrolled in the study. When DRT or CME or both were concomitant with serous detachment, then the eye was admitted to the SRD group.^[12]^

CMT was defined as the distance from the inner border of the RPE layer to the inner side of the strong reflective zone of the retinal epithelium through the central macular recess. ELM and EZ disruption were evaluated specifically in the region within 1 mm of the central fovea and were defined as places where the original medium to high reflectivity disrupts the integrity and continuity of the ELM or ellipsoid band by OCT. DRIL was defined by the inability to identify and demarcate any boundaries of the ganglion cell layer, inner plexiform layer, inner nuclear layer, and outer plexiform layer in the central 1 mm of the macular region.

#### 2.3.2. Intravitreal aflibercept treatment.

All eyes received 0.05 mL/2 mg aflibercept intravitreal injection in the operating room using a strict aseptic technique. After giving topical anesthetic drops and sterilizing by povidone-iodine, aflibercept (0.05 mL/2 mg) was injected through the vitreous cavity 3.5 to 4.0 mm posterior to the ciliary flat using a 30-gauge needle. Each patient received monthly repeat injections 5 times. Topical antiglaucoma medication was started in patients with IOP ≥ 22 mm Hg at all follow-up times.

### 2.4. Data analysis

For data analysis, the Statistical Package for Social Sciences (version 22.0; IBM, Armonk, NY) was used. Numeric variables are stated in terms of mean standard deviation, while categorical variables are expressed in terms of frequencies and percentages. BCVA measurements were converted to logMAR equivalents for statistical analysis. Paired *t* tests were employed to analyze preinjection and postinjection BCVA and the mean CMT in each subgroup. One-way analysis of variance tests (Tukey post hoc test) were performed to assess variations among the 3 groups. The risk factors affecting the response of DME patients to aflibercept treatment were analyzed using binary logistic regression. Statistical significance was determined at *P* < .05 (2-sided).

## 3. Results

### 3.1. Baseline characteristics and patient demographics

A total of 130 eyes from 95 consecutive patients (47 males, 48 females) were included. Table 1 summarizes patient demographics and features. There were 40 eyes with 28 patients (male 13, female 15), average age (56.73 ± 7.40) in the DRT group and 46 eyes with 35 patients (male 16, female 19), average age (55.54 ± 6.15) in the CME group. There were 44 eyes with 29 patients (male 18, female 14), average age (55.81 ± 5.65) in the SRD group. The baseline characteristics were not statistically significant among the 3 groups; these characteristics comprised age (*P* = .947), sex (*P* = .791), duration of diabetes (*P* = .885), HbA1c level (*P* = .604), IOP (*P* = .189), nonproliferative diabetic retinopathy stage (*P* = .093), baseline BCVA in logMAR units (*P* = .305), and baseline CMT (*P* = .114). Among the 3 groups, the baseline OCT features of DRIL, ELM disruption, and EZ disruption were statistically significant (*P* = .027, *P* < .001, and *P* < .001, respectively).

**Table 1:** Baseline characteristics of patients with diabetic macular edema.

	DRT	CME	SRD	*X*^2^/*F* value	*P* value
Eyes*	40	46	44	0.278	.87
Sex* (male/female)	13/15	16/19	18/14	0.237	.791
Age† (yr)	56.73 ± 7.40	55.54 ± 6.15	55.81 ± 5.65	0.055	.947
Duration of diabetes† (yr)	12.75 ± 7.19	13.58 ± 7.59	14.17 ± 6.28	0.123	.885
HbA1C† (%)	7.65 ± 0.33	8.02 ± 0.71	7.81 ± 0.74	0.522	.604
IOP† (mm Hg)	16.33 ± 2.87	15.56 ± 1.88	17.78 ± 2.73	1.786	.189
ELM disruption* (%)	13 (32.5%)	30 (65.2%)	33 (75%)	16.922	<.001
EZ disruption* (%)	11 (27.5%)	28 (60.9%)	34 (77.3%)	21.725	<.001
NPDR* (%)				7.965	.093
Mild NPDR	23 (57.5%)	10 (25%)	7 (17.5%)		
Moderate NPDR	18 (39.1%)	13 (28.3%)	15 (32.6%)		
Severe NPDR	14 (31.8%)	11 (25%)	19 (43.2%)		
DRIL* (%)	25 (62.6%)	37 (80.4%)	38 (86.8%)	7.216	.027

Categorical variables are shown as numbers (%), and continuous variables are presented as the means ± standard.

CME = cystoid macular edema, DRIL = disorganization of the inner retinal layers, DRT = diffuse retinal thickness, ELM = external limiting membrane, EZ = ellipsoid zone, HbA1C = glycated hemoglobin, IOP = intraocular pressure, NPDR = non-proliferative diabetic retinopathy, SRD = serous retinal detachment.

* Chi-square test; † One-way analysis of variance (ANOVA) (post hoc Tukey).

### 3.2. Changes in mean BCVA

**Figure 3.**
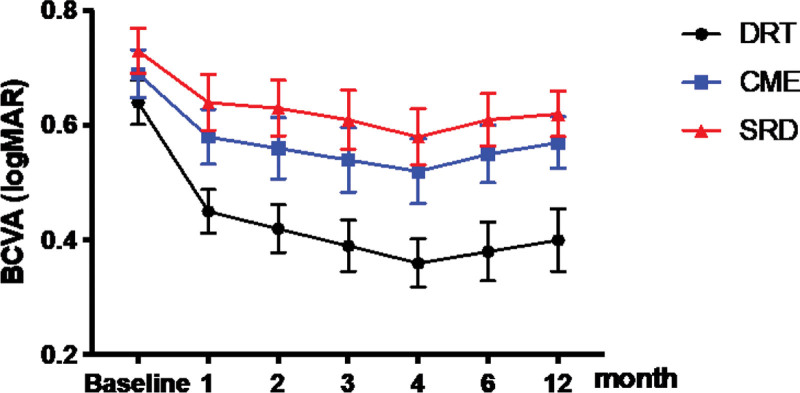
Change in the mean BCVA over time after aflibercept injection. At 4 months, the DRT group (0.36 ± 0.13) showed a better BCVA than the CME (0.51 ± 0.19) and SRD (0.58 ± 0.16) groups (*P* = .017). There were significant differences in the mean BCVA value changes among the 3 groups (*P* = .017). At 12 months, the DRT group (0.40 ± 0.17) showed a better BCVA than the CME (0.57 ± 0.15) and SRD (0.62 ± 0.13) groups (*P* = .018). There were significant differences in the mean BCVA value changes among the 3 groups (*P* = .021). BCVA = best corrected visual acuity, CME = cystoid macular edema, DRT = diffuse retinal thickening, SRD = serous retinal detachment.

Table 2 and Figure 3 describe the changes in BCVA for different OCT types at baseline and after intravitreal aflibercept injection. At 4 months, the DRT group (0.36 ± 0.13) showed a better BCVA than the CME (0.51 ± 0.19) and SRD (0.58 ± 0.16) groups (*P* = .017). Eyes of the DRT type showed a significant increase in BCVA compared with baseline (0.64 ± 0.12) (*P* = .011). Eyes of the CME type and SRD type had a significantly better BCVA than those at baseline (*P* = .023, *P* = .035). Meanwhile, there were significant differences in the mean BCVA value changes between the 3 groups (*P* = .017), and the DRT group had the greatest improvement in visual acuity.

**Table 2:** The changes in BCVA at baseline and after intravitreal aflibercept injection for different OCT types.

	DRT (LogMAR)	CME (LogMAR)	SRD (LogMAR)	*F* value	*P* value
Baseline (1st inj.)	0.64 ± 0.12	0.69 ± 0.14	0.73 ± 0.13	1.362	.273
1-mo (2nd inj.)	0.45 ± 0.12*	0.58 ± 0.16	0.64 ± 0.16	4.451	.022
Changes	−0.19 ± 0.15	−0.11 ± 0.19	−0.09 ± 0.18	3.905	.027
2-mo (3rd inj.)	0.42 ± 0.13*	0.56 ± 0.18	0.63 ± 0.16	3.912	.024
Changes	−0.22 ± 0.15	−0.13 ± 0.2	−0.1 ± 0.19	4.387	.016
3-mo (4th inj.)	0.39 ± 0.14*	0.53 ± 0.19	0.61 ± 0.16	5.988	.007
Changes	−0.25 ± 0.13	−0.14 ± 0.19	−0.1 ± 0.18	9.033	.004
4-mo (5th inj.)	0.36 ± 0.13*	0.51 ± 0.19*	0.58 ± 0.16*	4.622	.017
Changes	−0.27 ± 0.14	−0.17 ± 0.2	−0.15 ± 0.19	8.196	.002
6-mo	0.38 ± 0.16*	0.55 ± 0.17	0.61 ± 0.15	8.455	.008
Changes	−0.25 ± 0.1	−0.14 ± 0.16	−0.12 ± 0.13	9.991	.001
12-mo	0.40 ± 0.17*	0.57 ± 0.15	0.62 ± 0.13	7.655	.018
Changes	−0.24 ± 0.18	−0.12 ± 0.18	−0.11 ± 0.16	4.469	.021

BCVA = best-corrected visual acuity, CME = cystoid macular edema, DRT = diffuse retinal thickness, inj = intravitreal injections, logMAR = logarithm of the minimum angle of resolution, OCT = optical coherence tomography, SRD = serous retinal detachment.

* *P* < .05, compared to each baseline, Paired *t* test. Three-group comparison, one-way analysis of variance (ANOVA) (post hoc Tukey).

At 12 months, the DRT group (0.40 ± 0.17) showed a better BCVA than the CME (0.57 ± 0.15) and SRD (0.62 ± 0.13) groups (*P* = .018). DRT type eyes showed a significant increase in BCVA compared with baseline (0.64 ± 0.12) (*P* = .018). Eyes of the CME type and SRD type had a slightly better BCVA at 12 months than at baseline (*P* = .062, *P* = .074). Meanwhile, there were significant differences in the mean BCVA value changes between the 3 groups (*P* = .021), and the DRT group had the greatest improvement in visual acuity.

### 3.3. Changes in mean CMT

**Figure 4.**
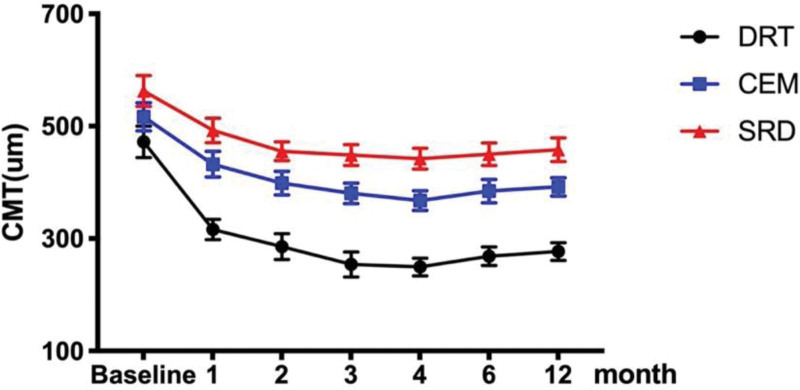
Change in the mean CMT over time after intravitreal aflibercept injection. At 12 months, the eyes of all 3 groups had lower CMT values than at baseline (*P* = .001, *P* = .004, *P* = .009). The reduction in CMT was more evident in the DRT group (277.16 ± 49.39 µm) than in the CME (392.23 ± 55.47 µm) and SRD (452.26 ± 65.64 µm) groups (*P* = .009). Meanwhile, there were significant differences in the changes in the reduction in CMT among the 3 groups (*P* = .022). CME = cystoid macular edema, CMT = central macular thickness, DRT = diffuse retinal thickening, SRD = serous retinal detachment.

Table 3 and Figure 4 are summarized and show the influence of intravitreal aflibercept on CMT based on different OCT types. At 12 months, the eyes of all 3 groups had lower CMT values than at baseline (*P* = .001, *P* = .004, *P* = .009). The reduction in CMT was more evident in the DRT group (277.16 ± 49.39 µm) than in the CME (392.23 ± 55.47 µm) and SRD (452.26 ± 65.64 µm) groups (*P* = .009). Meanwhile, there were significant differences in the changes in the reduction in CMT among the 3 groups (*P* = .022).

**Table 3:** Changes in CMT at baseline and after intravitreal aflibercept injection for different OCT types.

	DRT	CME	SRD	*F* value	*P* value
	CMT (µm)	CMT (µm)	CMT (µm)		
Baseline (1st inj.)	472.4 ± 87.75	516.64 ± 84.44	562.73 ± 85.73	2.349	.115
1-mo (2nd inj.)	316.3 ± 55.71*	432.4 ± 76.31*	492.7 ± 68.04*	3.914	.032
Changes	−156.1 ± 70.16	−84.18 ± 51.66	−70.46 ± 50.62	11.986	.001
2-mo (3rd inj.)	285.7 ± 72.44*	398.82 ± 70.96*	455.36 ± 52.29*	5.474	.01
Changes	−186.7 ± 70.51	−117.82 ± 66.38	−107.37 ± 82.10	4.512	.015
3-mo (4th inj.)	254.1 ± 69.34*	380.64 ± 61.59*	448.82 ± 57.56*	8.271	.007
Changes	−218.3 ± 63.52	−136.0 ± 76.43	−113.91 ± 85.38	8.298	.006
4-mo (5th inj.)	249.6 ± 49.08*	367.69 ± 58.31*	442.06 ± 57.92*	8.924	.004
Changes	−222.8 ± 72.61	−158.95 ± 81.23	−120.67 ± 83.04	7.912	.017
6-mo	268.9 ± 51.36*	384.7 ± 69.72*	450.38 ± 62.65*	6.991	.003
Changes	−203.5 ± 82.45	−121.94 ± 81.26	−101 35 ± 83.27	12.182	.001
12-mo	277.2 ± 49.39*	392.23 ± 55.47*	452.26 ± 65.64*	8.269	.009
Changes	−195.2 ± 71.49	−124.41 ± 76.25	−110.47 ± 81.56	4.451	.022

CME = cystoid macular edema, CMT = central macular thickness, DRT = diffuse retinal thickness, inj = intravitreal injections, OCT = optical coherence tomography, SRD = serous retinal detachment.

* *P* < .05; compared to each baseline, Paired *t* test. Three-group comparison, one-way analysis of variance (ANOVA) (post hoc Tukey).

### 3.4. The effective and ineffective groups

Table 4 summarizes the different clinical data for the effective and ineffective groups. According to the visual acuity improvement, the patients with DME were separated into 2 groups: effective and ineffective. The differences in age, duration of diabetes, IOP, HbA1c, baseline CMT, and baseline BCVA between the effective and ineffective groups were not statistically significant (*P* = .766, *P* = .724, *P* = .580, *P* = .831, *P* = .075, *P* = .059). However, the differences in EZ disruption and ELM disruption between the effective group and the ineffective group were statistically significant (*P* < .001, *P* < .001). Additionally, the difference in DRIL between the effective group and the ineffective group was statistically significant (*P* < .001).

**Table 4:** The different clinical data for the effective and ineffective groups.

	Effective group	Ineffective group	*X*^2^/*t* value	*P* value
DME types*	–	–	7.371	.026
DRT (%)	32 (80%)	8 (20%)		
CME (%)	30 (65.2%)	16 (34.8%)		
SRD (%)	15 (34.1%)	29 (65.9%)		
Age† (yr)	55.96 ± 8.52	58.24 ± 9.17	0.302	.766
Duration of diabetes† (yr)	12.25 ± 7.14	14.89 ± 6.54	0.354	.724
HbA1C† (%)	7.62 ± 0.34	7.98 ± 0.75	0.216	.831
IOP † (mm Hg)	17.25 ± 1.89	19.21 ± 1.92	0.564	.580
ELM disruption* (%)	29 (37.8%)	44 (82.2%)	21.893	<.001
EZ disruption* (%)	26 (33.5%)	41 (78.7%)	20.108	<.001
DRIL* (%)	27 (35.1%)	40 (75.5%)	20.521	<.001
Baseline CMT† (µm)	513.72 ± 79.68	547.06 ± 83.24	2.010	.075
Baseline BCVA† (logMAR)	0.63 ± 0.21	0.68 ± 0.19	2.162	.059

Categorical variables are shown as numbers (%), and continuous variables are presented as the means ± standard.

BCVA = best corrected visual acuity, CME = cystoid macular edema, CMT = central macular thickness, DME = diabetic macular edema, DRIL = disorganization of the inner retinal layers, DRT = diffuse retinal thickness, ELM = external limiting membrane, EZ = ellipsoid zone, HbA1C = glycated hemoglobin, IOP = intraocular pressure, logMAR = logarithm of the minimum angle of resolution, SRD = serous retinal detachment.

* Chi-square test; † One-way ANOVA (post hoc Tukey).

### 3.5. Analysis of factors affecting the therapeutic effect of aflibercept

The risk factors affecting the response of DME patients to aflibercept treatment were analyzed using binary logistic regression. In this analysis, DME type, DRIL, EZ disruption, and ELM disruption were used as independent variables, while visual acuity improvement was used as the dependent variable; the analysis is summarized in Table 5.

**Table 5:** Risk factors affecting the response of DME patients to aflibercept treatment.

	*β*	SE	Wald *x*^2^ value	*P* value	OR value	95% CI
DME-types	4.256	1.519	7.477	.006	3.854	1.245–6.818
ELM disruption	4.027	1.391	8.362	.004	5.980	3.651–56.911
EZ disruption	6.555	1.759	14.043	.001	8.581	2.766–65.424
DRIL	5.908	1.603	10.391	.002	7.022	3.244–61.778

Binary logistic regression. *P* < .05 was considered statistically significant.

CI = confidence interval, DME = diabetic macular edema, DRIL = disorganization of the inner retinal layers, ELM = external limiting membrane, EZ = ellipsoid zone, OR =odds ratio, SE = standard error.

The variables were coded as follows: DME type (DRT = 0, CME = 1, SRD = 2), DRIL (yes = 1, no = 2), EZ disruption (yes = 1, no = 0), ELM disruption (yes = 1, no = 0), and visual acuity improvement (yes = 1, no = 0). The results showed that DME type, DRIL, EZ disruption, and ELM disruption were independent factors predicting the effect of aflibercept treatment in patients with DME (*P* = .006, *P* = .002, *P* = .004, and *P* = .001, respectively).

### 3.6. Injection times among the 3 groups

The average number of injections in the DRT, CME, and SRD groups was 5.16 ± 1.14, 5.65 ± 1.22, and 6.34 ± 1.36, respectively, and the difference in the number of injections among the 3 groups was statistically significant (*P* = .043).

### 3.7. Adverse events

There were no adverse events related to intravitreal injections during the research period, including excessive IOP, endophthalmitis, retinal detachment or vomiting, headache, anemia, and cardiovascular and cerebrovascular illnesses.

## 4. Discussion

In this study, we compared the anatomical and functional outcomes of different OCT classifications with DME after intravitreal aflibercept treatment. We found that the BCVA and CMT in the DRT, CME, and SRD groups were better than at baseline to some extent at 1, 2, 3, 4, 6, and 12 months, suggesting that the effect of aflibercept in treating DME was confirmed. This may be due to the ability of aflibercept to bind VEGF-A, placental growth factor, and other factors in multiple targets, which can reduce vascular permeability and have a good anti-neovascular effect, thus achieving a therapeutic effect on DME. Moreover, a significant discovery from this study is that the DRT group had the most visual acuity benefit, the most significant CMT reduction, and the fewest injections compared with the other groups.

To our knowledge, the efficacy and prognosis of different types of DME patients treated with anti-VEGF drugs are still controversial. Previous studies have demonstrated that intravitreal aflibercept treatment is likely to be more effective than ranibizumab in eyes with DME.^[8,13]^ In the current literature,^[14–16]^ there are some studies reported on the DRT type that found a greater relationship between improvement in visual acuity and reduction in macular thickness compared to the other 2 types, which is similar to the results of our study. Shimura et al^[17]^ also showed that the DRT type had thinner CMT and higher baseline BCVA than the other types, although the anatomic and visual results for anti-VEGF were not statistically significant.

However, in a retrospective study of 56 eyes of DME patients, Roh et al^[18]^ confirmed that the CME type had better improvement in CMT and BCVA after anti-VEGF injection than patients with diffuse macular edema. Koytak et al^[19]^ also demonstrated the efficacy of intravitreal bevacizumab in 92 eyes with different types of DME, and they suggested that there was no significant difference in visual acuity for the DRT type, but the CMT changes in the DRT group were relatively lower than those in the CME and SRD groups. In several recent studies,^[20–22]^ the SRD type appeared to have greater visual and anatomical advantages. These controversial findings may be consistent with incomplete agreement in DME types (attribution of the mixed type and vitreoretinal intersection interface abnormalities inclusion), different anti-VEGF drugs (bevacizumab, ranibizumab, aflibercept), different treatment regimens, the duration of follow-up and the sample size.

In this study, the different types of DME were found to be independent risk factors for the outcome of aflibercept treatment in patients with DME. This may be because the DRT type is regarded as an early stage of ME development, in which the main pathological mechanism is an increase in VEGF levels and the occurrence of inner blood–retinal barrier damage caused by Müller cell abnormalities, which increases the permeability of blood vessels without cystic changes. Therefore, the recovery of BCVA and CMT after treatment is better.^[23]^ In patients with CME, the influence of VEGF and inflammatory factors increased Müller cell damage and led to necrosis, resulting in fluid spreading to the central region and cystic cavity and further aggravation of the inner blood–retinal barrier damage. Moreover, except for VEGF, other inflammatory cytokines and prostaglandins play an important role in CME in patients with diabetes, and anti-VEGF alone may have a limited effect on the treatment of CME.

Patients with SRD are mainly affected by persistently high levels of retinal inflammatory factors and hypoxia-ischemia, which involve RPE function and restrict fluid transfer, causing fluid to accumulate in the outer layers of the retina and having a greater impact on the EZ and the ELM.^[24]^ It is believed that the pathological mechanism of SRD patients is not only an increase in VEGF levels but also abnormal RPE function. Therefore, although macular edema in SRD patients is reduced after aflibercept treatment, the recovery of BCVA is still limited after treatment, which increases the injection rate.

Moreover, we found that the EZ disruption ratio and ELM disruption ratio were highest in the SRD type. Further research also revealed that EZ and ELM disruption is an independent risk factor for the effect of aflibercept treatment in patients with DME. Similar conclusions were reached for the efficacy of other anti-VEGF drugs (ranibizumab/bevacizumab) in the treatment of DME, in which the disruption of the ELM and EZ was associated with worse baseline and poor posttreatment visual acuity.

The ELM is thought to be a linear confluence of cellular junctional complexes between Müller cells and photoreceptors, acting as a macromolecular barrier. The EZ represents the integrity of photoreceptors. Suciu et al^[25]^ reported that EZ and ELM interruption may increase VEGF levels and retinal fluid accumulation, which may increase CMT levels, diminish visual impairment, and contribute to the development of DME. Meanwhile, studies have confirmed that although EZ and ELM interruption can be recovered by anti-VEGF therapy, the effect is limited.^[26]^

Currently, DRIL is thought to result from disruption of the visual pathway from photoreceptor cells to ganglion cells, which has been confirmed to be an important biomarker of DME and can predict visual outcomes.^[27]^ In the present study, we demonstrated that the presence of DRIL is an independent factor predicting the effect of aflibercept treatment in patients with DME, which is consistent with the results of most domestic and international studies. We also found that the prevalence of DRIL was lowest in the DRT type, suggesting that the visual pathway may be more complete in this type than in other types.

However, there are some limitations of this clinical study. First, the follow-up period was short, only 12 months. Second, our sample size was small. Finally, we did not use blood flow OCT for quantitative measurements of retinal blood flow signals. For this part of the imaging parameters, the changes and correlation of these imaging parameters during anti-VEGF treatment in different strains of DME will be analyzed in future studies.

In conclusion, our results show that aflibercept can bring about visual improvement and anatomical changes in every pattern of DME. We also demonstrated that different patterns may respond differently to this drug. The DRT type has a better response than the other 2 types. Moreover, DME type, DRIL, EZ disruption, and ELM disruption are independent factors predicting the effect of aflibercept treatment in patients with DME. Therefore, the different classifications of macular edema by OCT may be an objective guide to predict the response to aflibercept injection in parents with DME.

## Author contributions

**Conceptualization:** Zheyao Gu.

**Data curation:** Zheyao Gu, Ting Xi.

**Investigation:** Chunyuan Zhang.

**Methodology:** Zheyao Gu, Guang Yang.

**Writing – original draft:** Zheyao Gu.
